# Physical activity and executive function in adolescents: Dual rumination pathways and mindfulness moderation

**DOI:** 10.1371/journal.pone.0357384

**Published:** 2026-09-01

**Authors:** Shimeng Wang, Mingyang Lu, Yang Yang, Bochun Lu, Yiwen Zhou, Shipeng Ding

**Affiliations:** 1 Institute of Sports Science, Nantong University, Nantong, China; 2 Department of Physical Education, Dankook University, Yongin, South Korea; 3 Department of Physical Education, Sun Yat-sen University, Guangdong, China; 4 College of Physical Education, Dalian University, Dalian, China; South China Normal University, CHINA

## Abstract

**Background:**

Late adolescence is a developmental period in which executive functions are relatively consolidated but remain sensitive to behavioral and environmental influences. Physical activity (PA) has been associated with executive-function outcomes, but the psychological factors that may be related to this association remain unclear. Objective: This study examined whether PA_total was associated with a reaction-time-based executive-function composite (EF_RT) through intrusive rumination (IR) and deliberate rumination (DR), and whether mindfulness moderated the PA_total → IR and IR → EF_RT associations.

**Methods:**

Late adolescents (N = 450; 266 males; aged 17–19 years) completed measures of PA, rumination, and mindfulness. EF_RT was derived from standardized RT-based indicators from computerized Flanker, 2-back, and More–Odd Shifting tasks. Lower EF_RT values indicate better RT-based task performance. Structural equation models were used to test parallel indirect associations and observed-variable interaction terms, with gender included as a covariate. Bias-corrected bootstrapping with 5,000 resamples was used to estimate indirect and conditional indirect associations.

**Results:**

PA_total showed significant positive total and direct associations with EF_RT, indicating that higher PA_total was associated with higher RT-based scores, that is, slower or less efficient RT-based task performance. Significant indirect associations were observed through both IR and DR. Mindfulness moderated the PA_total → IR and IR → EF_RT associations, and the conditional indirect association through IR varied across levels of mindfulness. Because EF_RT is a reaction-time-based composite, positive coefficients involving EF_RT indicate higher RT-based scores, reflecting longer reaction times or larger RT costs, rather than better EF ability.

**Conclusions:**

PA_total, rumination, mindfulness, and EF_RT were interrelated in late adolescents. The findings should be interpreted as cross-sectional associations involving RT-based executive-function performance, including an unexpected positive PA_total–EF_RT association indicating slower or less efficient RT-based performance, rather than as evidence of causal effects on EF ability.

## 1. Introduction

Adolescence (10–19 years) is the developmental stage between childhood and adulthood [1]. It is a period of rapid cognitive development and increasing vulnerability to behavioral and emotional influences, making cognitive functioning during this stage an important topic of research [2]. Executive function (EF), as a higher-order cognitive function, refers to a set of cognitive processes required to execute complex and goal-directed operations, including three core components: inhibitory control, working memory, and cognitive flexibility [3]. EF plays an essential role in adolescents’ academic performance, socio-emotional functioning, and adaptive behavior [4]. Therefore, identifying effective strategies to enhance adolescents’ EF has become an important research focus. The present study specifically focuses on late adolescents (17–19 years), a developmental stage in which core executive functions are more consolidated than in earlier adolescence but still show developmental plasticity and remain sensitive to behavioral and environmental experiences [5–7].

EF during adolescence is influenced by various environmental and social factors. Among these factors, physical activity (PA) has been associated with cognitive function and EF-related outcomes in children and adolescents, although the strength and consistency of these associations may vary according to activity type, intensity, timing, and cognitive demands [5,8,9]. In addition, PA is relatively low-cost, easy to implement, has minimal side effects, and can be readily integrated into educational or intervention settings, making it a feasible strategy for supporting adolescents’ cognitive functioning. Previous studies generally explain the association between PA and EF from two perspectives. On the one hand, PA may be related to EF through physiological and neurocognitive mechanisms involving brain regions and networks associated with executive control. For example, regular physical activity has been linked to structural and functional brain changes involving regions and networks closely related to EF, including prefrontal and frontoparietal systems [8–10]. On the other hand, complex physical activities often involve higher cognitive demands and require greater allocation of cognitive resources [5]. Compared with simple physical activities, complex activities require individuals to process dynamic information, maintain attention, and flexibly adapt to changing situations [11, 12]. For instance, during a football game, players must continuously monitor the positions of teammates and opponents while adjusting their strategies in response to rapidly changing game situations [13]. Such cognitively engaging activities may activate brain regions related to EF and support EF-related cognitive processes [14]. Although existing research has extensively examined the association between PA and EF from physiological perspectives, relatively fewer studies have explored the psychological mechanisms underlying this relationship. However, examining PA and EF solely from a physiological perspective may provide an incomplete understanding of this association. Some scholars have proposed a PA–Cognitive Function Mediation Model, suggesting that psychological factors may also play an important role in linking PA and cognitive outcomes [15,16]. Compared with purely physiological mechanisms, psychological processes may better reflect individuals’ cognitive functioning in real-life social contexts. Therefore, examining psychological factors related to the association between PA and EF may provide a more comprehensive understanding of how PA is linked to cognitive functioning and may offer preliminary insights for future intervention research.

One psychological process that may help explain the association between PA and EF is rumination. Rumination reflects repetitive cognitive processing that is closely linked to affect regulation, attentional control, and the allocation of executive resources. It is generally regarded as a multidimensional cognitive process related to both EF and psychological well-being [17,18]. Early research commonly conceptualized rumination as a maladaptive cognitive style in which individuals repeatedly focus on negative emotions and their causes, meanings, and consequences without adopting effective coping strategies when facing stress or adverse events [19]. However, subsequent research has suggested that rumination may include both maladaptive and adaptive components. Maladaptive rumination, often referred to as intrusive rumination (IR), is characterized by involuntary and repetitive negative thoughts that may trigger negative emotional responses and interfere with goal-directed behavior. In contrast, adaptive rumination, commonly referred to as deliberate rumination (DR), involves more reflective and purposeful cognitive processing that may facilitate problem-solving and goal pursuit [20]. According to Papageorgiou and Wells [21], these two forms of rumination may exert different influences on individuals’ emotional states, goal-directed behavior, and self-evaluation. IR may consume limited cognitive resources and interfere with executive functioning by maintaining negative emotional states and persistent intrusive thoughts [17,22]. In contrast, DR may increase individuals’ sensitivity to goal-related information and motivate them to reflect on the causes of difficulties and potential strategies for goal attainment [23]. However, its relationship with EF-related outcomes may depend on whether such reflection remains constructive or becomes cognitively demanding.

Research on the development and mechanisms of rumination suggests that both biological and environmental factors may increase individuals’ tendency toward ruminative thinking [24]. Building on this perspective, several studies have examined the association between PA and rumination in specific populations. For example, studies have reported that regular PA is associated with reduced IR among inpatients with mental disorders [25]. PA has also been linked to emotional regulation and psychological well-being, which may be relevant to lower ruminative thinking [26–29]. Evidence from other populations provides similar indications. For instance, research on postmenopausal women has shown that individuals who maintain higher levels of physical activity exhibit lower rumination-related reactivity and better recovery from acute stress [30]. Moreover, some studies suggest that the duration of PA interventions may influence their association with rumination. For example, short-term PA interventions (e.g., 30 minutes) may not directly reduce rumination but may facilitate emotional recovery processes [31], whereas longer-term PA interventions have been associated with reductions in ruminative thinking [32]. Taken together, these findings from specific populations suggest a potential association between PA and rumination. However, most existing studies have focused on clinical or special populations. Investigating this association in non-clinical populations, particularly among adolescents, is equally important. Clarifying the relationship between PA and rumination during adolescence may provide valuable evidence for developing preventive strategies and promoting healthy lifestyles aimed at improving adolescents’ mental health.

Within a cognitive–affective processing framework, mindfulness is defined as an attentional mode characterized by present-moment awareness and nonjudgmental acceptance [33,34]. Through a process described as monitoring plus acceptance, mindfulness facilitates adaptive processing of both internal and external cues. Its mechanisms have been conceptualized as decentering or reperceiving and reduced cognitive reactivity, which may weaken automatic negative processing and the associated consumption of cognitive resources [35]. Evidence from neural and cognitive research further indicates that mindfulness is associated with enhanced top-down attentional control and stronger coupling of executive networks, helping individuals maintain proactive control during cognitive tasks and safeguard limited executive resources [36–38]. On this basis, the present study conceptualizes mindfulness as a moderator rather than as a mediator. This choice was made because the current model does not assume that PA first changes adolescents’ dispositional mindfulness, which would be required for a mediation pathway. Instead, mindfulness is treated as an attentional and self-regulatory disposition that may condition how adolescents process physical-activity-related affective experiences and ruminative thoughts. In this sense, mindfulness may alter the strength of the PA → IR association and the IR → EF_RT association. On the one hand, PA may be associated with affect regulation and attentional engagement; individuals with higher levels of mindfulness may process physical-activity-related bodily and affective experiences in a less elaborative manner, which may strengthen the negative association between PA and IR [39]. On the other hand, mindfulness may condition the association between IR and EF-related task performance. Research in student populations suggests that mindfulness moderates the relationship between rumination and its perceived uncontrollability, indicating that higher levels of mindfulness may reduce the likelihood that rumination develops into a persistent negative cognitive cycle and consequently limit its consumption of cognitive resources [40]. Accordingly, the present study positions mindfulness as a moderator at two key loci (PA → IR; IR → EF_RT), generating conditional indirect associations within a moderated mediation framework.

In sum, converging evidence suggests that PA is associated with EF in adolescents. Rumination has also been closely linked to EF, and PA may be related to lower levels of maladaptive rumination. Building on this literature, rumination may be involved in the association between PA and EF-related task performance. However, empirical tests of these potential mechanisms in youth remain limited, highlighting the need for further investigation. Accordingly, the present study first tests a parallel mediation model in which PA is associated with EF_RT through two rumination pathways: intrusive rumination (IR) and deliberate rumination (DR). This model is then extended into a moderated mediation framework by examining whether mindfulness moderates two theoretically important paths, namely the PA → IR path and the IR → EF_RT path. Thus, the moderated mediation model should be understood as a conditional extension of the initial parallel mediation model, rather than as a separate or competing analytical approach. By examining these relationships, the present study aims to characterize psychological association patterns linking PA, rumination, mindfulness, and EF_RT, and to provide preliminary evidence that may inform future longitudinal and intervention research on adolescents’ cognitive and mental health.

## 2. Materials and methods

### 2.1. Participants and data collection

The study adopted a convenience sampling approach, recruiting late adolescents from six universities located in Jiangsu, Anhui, and Liaoning provinces in China. Data were collected between March 1, 2025 and July 1, 2025. In total, 530 questionnaires were obtained. After data screening, responses were excluded if (1) any item was missing or (2) there was evidence of straight-lining (i.e., all identical responses or a regular response pattern). This resulted in 450 valid questionnaires (valid response rate = 85%). Among the respondents, 266 were male (59.1%) and 184 were female (40.9%). Participants were aged between 17 and 19 years (M = 18.29, SD = 0.63). This study was approved by the Ethics Committee of the Medical College of Nantong University (Approval No.: TD-2024–109). All participants provided written informed consent prior to participation. For participants under the age of 18, written informed consent was obtained from their parents or legal guardians. All procedures were conducted in accordance with the Declaration of Helsinki.

### 2.2. Measures

#### 2.2.1. Physical Activity.

Physical activity was assessed using the Physical Activity Rating Scale–3 (PARS-3) developed by Liang [41]. The scale consists of three items assessing exercise intensity, exercise duration (time), and exercise frequency, with each item rated on a five-point scale. According to the standard scoring procedure proposed by Liang [41], the continuous PA total score was calculated as follows: PA_total = Intensity × (Time − 1) × Frequency. The resulting score ranges from 0 to 100, with higher scores indicating higher levels of physical activity. Based on conventional classification criteria, PA levels can also be categorized as low (≤19), moderate (20–42), and high (≥43). In the present study, the continuous PARS-3 total score (PA_total) was used in the primary correlation and SEM analyses, whereas the categorical PA level was retained only for descriptive purposes.

#### 2.2.2. Rumination.

Rumination was assessed using the Chinese version of the Event-Related Rumination Inventory (C-ERRI), originally developed by Cann et al. [42] and adapted for Chinese populations by Dong et al. [43]. The C-ERRI includes two dimensions: intrusive rumination (IR) and deliberate rumination (DR), each consisting of 10 items, resulting in a total of 20 items. Participants rated the frequency of ruminative thoughts experienced during the past two weeks using a four-point Likert scale ranging from 1 (“never”) to 4 (“always”), with higher scores indicating greater levels of rumination.

The C-ERRI was selected because the present study specifically aimed to distinguish between intrusive and deliberate forms of rumination. These two dimensions corresponded directly to the proposed dual rumination pathways in the theoretical model. However, because the ERRI was originally developed to assess rumination following a specific stressful or traumatic event, its use in the present non-clinical adolescent sample without a single event-specific reference should be interpreted cautiously. In this study, the C-ERRI scores were therefore interpreted as ERRI-based indicators of intrusive and deliberate ruminative thinking over the recent two-week period, rather than as event-specific post-traumatic rumination. This measurement issue is acknowledged as a limitation. In the present study, the scale demonstrated good internal consistency, with Cronbach’s alpha coefficients of 0.919 for IR and 0.923 for DR.

#### 2.2.3. Executive function.

Executive function (EF) was assessed using a web-based computerized neuropsychological assessment system. Participants logged into the system using preassigned accounts and passwords to complete three cognitive tasks: the Flanker task, the 2-back task, and the More–Odd Shifting task, corresponding to inhibitory control, working memory, and cognitive flexibility, respectively. This assessment system has been applied and validated in previous studies and is suitable for use with late adolescents. It supports group-based administration, local operation, and automatic recovery after network interruptions without affecting the testing procedure or data integrity [44, 45].

During testing, participants were instructed to respond as quickly and accurately as possible. For each task, RT-based performance indicators were extracted according to the scoring rules of the computerized assessment system. The Flanker task provided an inhibitory-control interference-cost index (IC_cost), calculated from the reaction-time difference between incongruent and congruent trials. The 2-back task provided a working-memory reaction-time index (WM_RT), and the More–Odd Shifting task provided a cognitive-flexibility reaction-time index (CF_RT). These indicators were not interpreted as accuracy scores or positively scored ability scores. Instead, they reflected RT-based task performance, with lower values indicating shorter reaction times or smaller RT costs and therefore better task performance.

Because the three EF indicators were on different raw scales and represented different task-specific RT-based metrics, they were not directly summed in their raw form. Instead, IC_cost, WM_RT, and CF_RT were standardized and averaged to form the EF_RT composite. The standardized indicators were not reverse-coded; therefore, higher EF_RT values indicate longer reaction times or larger RT costs, whereas lower EF_RT values indicate better RT-based task performance. Accuracy rates for the three EF tasks were also recorded and are reported in Supplementary S1 Table to help evaluate whether the RT-based indicators were interpreted under acceptable task-performance conditions.

This composite approach was used to provide an overall summary of RT-based performance across inhibitory control, working memory, and cognitive flexibility. However, because each EF domain was represented by a single task indicator, EF_RT should not be interpreted as a latent EF factor or as a complete measure of the unity-and-diversity structure of executive function. Therefore, we retained the standardized RT-based composite as a pragmatic summary index, while reporting the three domain-specific RT-based indicators descriptively.

#### 2.2.4. Mindfulness.

Mindfulness was assessed using the Five Facet Mindfulness Questionnaire (FFMQ), originally developed by Baer et al. [46] and adapted for Chinese populations by Deng et al. [47]. The scale consists of 39 items rated on a five-point Likert scale ranging from 1 (“not at all true”) to 5 (“completely true”). The FFMQ measures five dimensions of mindfulness: observing, describing, acting with awareness, nonjudging of inner experience, and nonreactivity to inner experience. The observing, describing, acting with awareness, and nonjudging subscales each contain eight items, whereas the nonreactivity subscale contains seven items. Higher total scores indicate higher levels of mindfulness. In the present study, the scale demonstrated excellent internal consistency, with a Cronbach’s alpha of 0.938.

### 2.3. Data analytic strategy

Structural equation modeling (SEM) was conducted using AMOS 26.0 with maximum likelihood estimation. The continuous PARS-3 total score (PA_total), calculated using the formula Intensity × (Time − 1) × Frequency, was used as the physical activity variable in the correlation, mediation, and moderated mediation analyses. The categorical PA level classification was not used as the predictor in the SEM models. Thus, the three-level PA category variable (1 = low, 2 = moderate, 3 = high) was not treated as a continuous predictor in any analysis. All correlation, mediation, and moderated mediation analyses were conducted using the original continuous PARS-3 total score (PA_total). Because PA_total was treated as a continuous score, Pearson correlations were used for the main correlation analyses. The significance level was set at α = .05.

For the moderated mediation analysis, interaction terms were constructed as observed product terms rather than latent variable interactions. Specifically, PA_total, intrusive rumination (IR), and mindfulness were mean-centered, and two product terms were computed: PA_total × mindfulness and IR × mindfulness. These product terms were then entered into the AMOS model as observed variables to test whether mindfulness moderated the PA_total → IR path and the IR → EF_RT path. No latent interaction terms or latent variable scores were used. Because AMOS does not automatically provide PROCESS-style conditional indirect effects for moderated mediation models, the conditional indirect effects were calculated from the estimated path coefficients after fitting the observed-variable interaction model. The conditional indirect association through IR was computed at three levels of mindfulness (M − 1 SD, M, and M + 1 SD). Specifically, the conditional PA_total → IR slope and the conditional IR → EF_RT slope were first estimated at each level of mindfulness, and the conditional indirect association was calculated as the product of these two conditional slopes.

Because mindfulness moderated both the PA_total → IR path and the IR → EF_RT path, the full conditional indirect association was calculated as the product of two moderator-dependent slopes: a(W) × b(W), where a(W) represents the conditional PA_total → IR slope and b(W) represents the conditional IR → EF_RT slope at a given level of mindfulness. Thus, the full conditional indirect association contains the joint product implied by the two moderated paths. To avoid implying a single linear index of moderated mediation, the values reported in Table 7 were treated as path-specific component indices. The a-path component index was calculated as the change in the conditional PA_total → IR slope per one-SD increase in mindfulness multiplied by the IR → EF_RT slope at mean mindfulness, whereas the b-path component index was calculated as the change in the conditional IR → EF_RT slope per one-SD increase in mindfulness multiplied by the PA_total → IR slope at mean mindfulness. The cross-product term was not reported as a separate Table 7 index, but the full conditional indirect associations in Table 8 were calculated as the product of the conditional PA_total → IR and IR → EF_RT slopes at each level of mindfulness.

Bias-corrected bootstrap procedures with 5,000 resamples were used to estimate 95% confidence intervals. For the conditional indirect effects in Table 8, a(W) and b(W) were recomputed and multiplied within each bootstrap sample, and the empirical distribution of these 5,000 products was used to construct the bias-corrected 95% confidence intervals. Conditional indirect associations were considered significant when the 95% confidence interval did not include zero. Multicollinearity was assessed after the mean-centered product terms were created. The results indicated no serious multicollinearity concern among the predictors and interaction terms (VIF = 1.01–1.52; Tolerance = 0.66–0.99), suggesting that the interaction estimates were not substantially distorted by collinearity.

**Fig 1:**
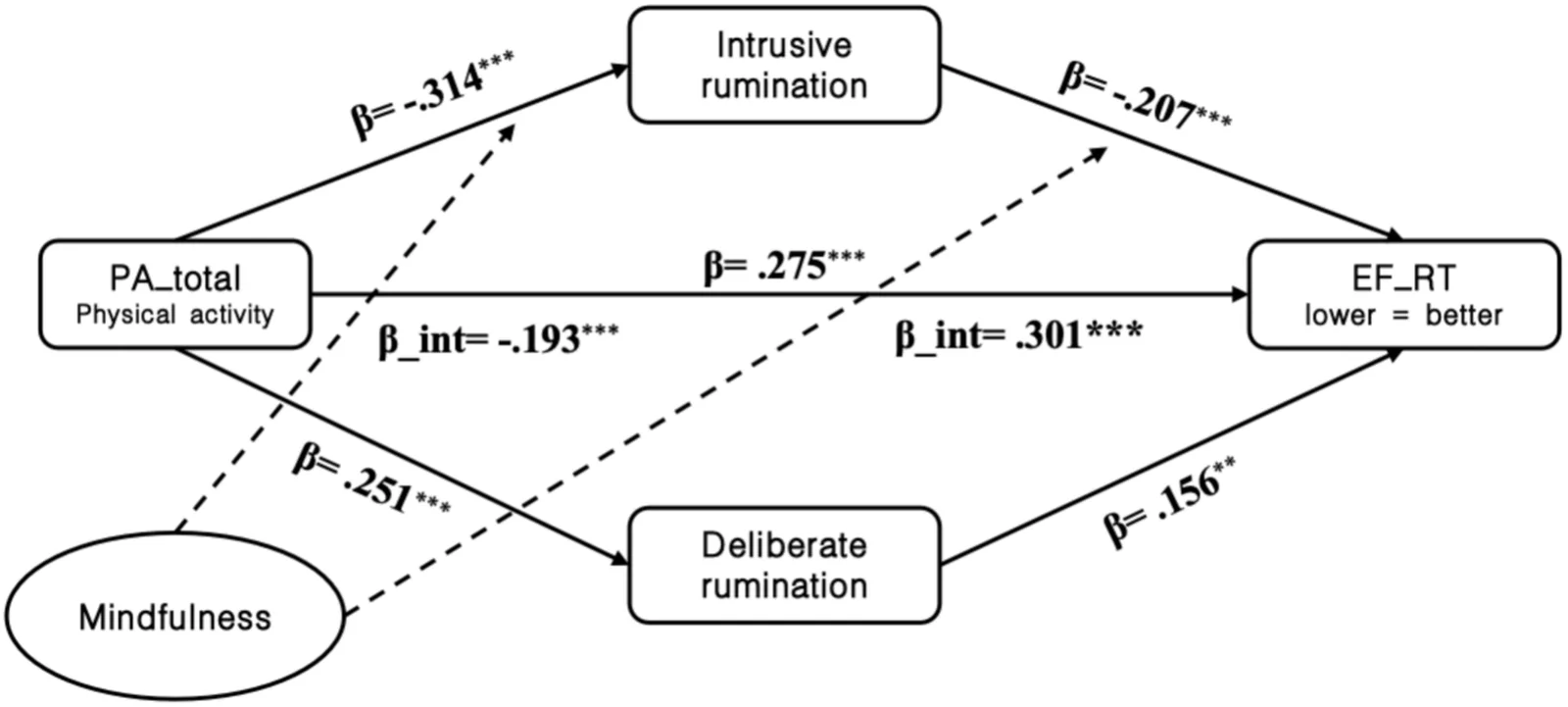
Moderated parallel mediation model linking PA_total to EF_RT via intrusive and deliberate rumination, with mindfulness moderating the PA_total → IR and IR → EF_RT paths. Standardized coefficients are shown. Dashed arrows indicate interaction effects. EF_RT = reaction-time-based executive-function composite; lower EF_RT values indicate better RT-based task performance. Gender was included as a covariate. **p < .01, ***p < .001. The PA_total → EF_RT path shown in the figure represents the direct path in the moderated mediation model, not a single unconditional total effect.

Given the observed gender differences in several key variables, gender was included as an observed covariate in the structural models. Specifically, gender was entered as a covariate in both the parallel mediation model and the moderated mediation model, and paths from gender to the endogenous variables were specified to adjust the estimated associations among PA_total, rumination, and EF_RT. Gender was also included as a covariate in the models reported in Table 6, Table 7, Table 8 and Fig 1.

A sensitivity power analysis was conducted using G*Power 3.1.9.7 to evaluate whether the sample size was adequate for detecting interaction effects in the moderated mediation model. Because G*Power does not directly estimate power for SEM-based moderated mediation, a linear multiple regression R^2^ increase model was used as an approximate sensitivity analysis for the interaction terms. With α = .05, power = .80, N = 450, two tested predictors, and nine total predictors, the smallest detectable incremental effect size was approximately f^2^ = 0.0216. This value is close to the conventional small-effect threshold, suggesting that the current sample provided reasonable sensitivity for detecting small incremental effects of the interaction terms.

## 3. Results

### 3.1. Reliability, validity, and the measurement model

A confirmatory factor analysis (CFA) was conducted for the multi-item self-report scales, including intrusive rumination, deliberate rumination, and mindfulness. All standardized factor loadings were significant and above the recommended threshold, ranging from 0.701 to 0.835. For rumination, factor loadings ranged from 0.701 to 0.772 for intrusive rumination (IR) and from 0.714 to 0.781 for deliberate rumination (DR). For the five facets of mindfulness—Observing, Describing, Nonjudging of Inner Experience, Nonreactivity to Inner Experience, and Acting with Awareness—factor loadings ranged from 0.709 to 0.819, 0.756 to 0.835, 0.725 to 0.816, 0.736 to 0.802, and 0.707 to 0.831, respectively (see Table 1). Convergent validity was supported, with average variance extracted (AVE) values ranging from 0.534 to 0.617 (≥ 0.50) and composite reliability (CR) values ranging from 0.909 to 0.928 (≥ 0.70) across constructs. Following the Fornell–Larcker criterion, the square roots of AVE exceeded the inter-construct correlations, supporting discriminant validity. Overall, the measurement model demonstrated satisfactory reliability and validity for the multi-item self-report constructs used in the subsequent analyses.

**Table 1:** Reliability and validity of the rumination and mindfulness scales.

Variable	Standardized factor loadings	AVE	CR
Intrusive rumination	0.701, 0.753, 0.739, 0.709, 0.710, 0.755, 0.724, 0.772, 0.717, 0.726	0.534	0.920
Deliberate rumination	0.730, 0.745, 0.719, 0.748, 0.714, 0.736, 0.724, 0.719, 0.781, 0.781	0.548	0.924
Observing	0.781, 0.808, 0.742, 0.709, 0.780, 0.748, 0.819, 0.747	0.589	0.920
Describing	0.786, 0.759, 0.757, 0.835, 0.756, 0.778, 0.790, 0.799	0.613	0.927
Nonjudging of Inner Experience	0.761, 0.775, 0.725, 0.727, 0.761, 0.795, 0.816	0.587	0.909
Nonreactivity to Inner Experience	0.761, 0.790, 0.802, 0.736, 0.796, 0.783, 0.754	0.600	0.913
Acting with Awareness	0.804, 0.775, 0.819, 0.776, 0.831, 0.707, 0.787, 0.777	0.617	0.928

Note. Values represent standardized factor loadings for the multi-item self-report scales. AVE = average variance extracted; CR = composite reliability. Recommended thresholds: factor loadings ≥ 0.70, AVE ≥ 0.50, and CR ≥ 0.70. PA_total and EF_RT were treated as observed variables and were therefore not included in this reflective measurement model.

PA_total and EF_RT were not included in this reflective measurement model because they were not multi-item reflective latent constructs. PA_total was a formula-based observed score calculated from the PARS-3 intensity, duration, and frequency items, rather than a latent construct indicated by multiple interchangeable reflective items. Therefore, factor loadings, AVE, and CR were not appropriate indices for evaluating PA_total.

Similarly, EF_RT was not a self-report latent scale. It was derived from computerized task-based RT indicators representing inhibitory control, working memory, and cognitive flexibility. Specifically, the Flanker task provided IC_cost, the 2-back task provided WM_RT, and the More–Odd Shifting task provided CF_RT. These three indicators were standardized and averaged to form the EF_RT composite. The indicators were not reverse-coded; therefore, lower EF_RT values indicate shorter reaction times or smaller RT costs and better RT-based task performance. The measurement and scoring procedures for PA_total and EF_RT are described in Sections 2.2.1 and 2.2.3, respectively.

Although EF_RT was not evaluated using AVE or CR, its measurement quality was examined in several ways. First, the three EF indicators were obtained from computerized tasks corresponding to the three core EF domains. Second, task accuracy was recorded and is reported in Supplementary S1 Table. The mean accuracy rates were 96.84% for the Flanker task, 81.93% for the 2-back task, and 93.76% for the More–Odd Shifting task, indicating that participants generally maintained acceptable accuracy levels while completing the RT-based EF tasks. These accuracy rates support the interpretation of the RT-based indicators under acceptable task-performance conditions, although accuracy-based indicators were not included in the main SEM models. Third, the three standardized EF indicators showed moderate positive intercorrelations (r = .496–.520), and the internal consistency of the three-indicator EF_RT composite was acceptable (Cronbach’s α = .759).

These results provided descriptive support for using EF_RT as a pragmatic RT-based summary index, while also indicating that it should not be interpreted as a latent EF factor or a complete measure of executive function.

### 3.2. Model fit

Model fit indices for the measurement model and the structural models are presented in Table 2. The measurement model demonstrated good fit, with χ²/df = 1.097, RMSEA = 0.015, SRMR = 0.036, CFI = 0.989, TLI = 0.989, NFI = 0.982, and IFI = 0.989. The gender-adjusted parallel mediation model also showed good fit, with χ²/df = 1.092, RMSEA = 0.014, SRMR = 0.032, CFI = 0.995, TLI = 0.995, NFI = 0.947, and IFI = 0.995. The complete moderated mediation model, which included the observed interaction terms PA_total × mindfulness and IR × mindfulness as well as gender as a covariate, likewise demonstrated acceptable to good fit, with χ²/df = 1.123, RMSEA = 0.017, SRMR = 0.039, CFI = 0.988, TLI = 0.988, NFI = 0.902, and IFI = 0.988. Overall, these fit indices supported the adequacy of the measurement and structural models for subsequent analyses [48].

**Table 2:** Fit indices for the measurement and structural models.

Model	χ²/df	RMSEA	SRMR	CFI	TLI	NFI	IFI
Measurement model	1.097	0.015	0.036	0.989	0.989	0.982	0.989
Parallel mediation model	1.092	0.014	0.032	0.995	0.995	0.947	0.995
Moderated mediation model	1.123	0.017	0.039	0.988	0.988	0.902	0.988

Note. χ²/df = chi-square divided by degrees of freedom; RMSEA = root mean square error of approximation; SRMR = standardized root mean square residual; CFI = comparative fit index; TLI = Tucker–Lewis index; NFI = normed fit index; IFI = incremental fit index. The parallel mediation model included gender as a covariate. The moderated mediation model included the observed interaction terms PA_total × mindfulness and IR × mindfulness, with gender included as a covariate.

### 3.3. Common method bias, descriptive statistics, and correlations

Common method variance (CMV) was examined using both Harman’s single-factor test and a common latent factor (CLF) approach. Because Harman’s single-factor test has limited sensitivity when used as the only diagnostic procedure, the results were interpreted cautiously and supplemented with a CFA-based CLF analysis. First, all self-report items were entered into an unrotated exploratory factor analysis. The analysis extracted four factors with eigenvalues greater than 1, and the first factor accounted for 38.92% of the total variance. Although this value was below the conventional 40% threshold, it was close to the cutoff and therefore was not considered sufficient evidence on its own to rule out CMV [49].

To further evaluate CMV, a CLF model was compared with the initial measurement model. The initial model showed good fit, χ²/df = 1.065, RMSEA = 0.012, SRMR = 0.036, CFI = 0.994, and TLI = 0.994. After adding the CLF, model fit changed only minimally, χ²/df = 1.042, RMSEA = 0.010, SRMR = 0.034, CFI = 0.997, and TLI = 0.996. The changes in fit indices were small (Δχ²/df = 0.023, ΔRMSEA = 0.002, ΔSRMR = 0.002, ΔCFI = 0.003, ΔTLI = 0.002), suggesting that CMV was unlikely to substantially distort the measurement model.

In addition, the study design partly reduced the risk of CMV because not all variables were obtained from the same self-report source. Specifically, EF_RT was derived from computerized reaction-time-based task indicators rather than from self-report questionnaires. Nevertheless, because PA_total, rumination, and mindfulness were assessed using self-report measures, CMV cannot be completely ruled out. This issue is therefore acknowledged as a methodological limitation.

Distributional assumptions were examined prior to model testing. The skewness and kurtosis values of the key variables were within ±1 in absolute value, indicating acceptable normality. Given the sample size (N = 450), the assumptions for maximum-likelihood (ML) estimation in SEM were considered acceptable. In the present analyses, physical activity was represented by the continuous PARS-3 total score (PA_total), calculated using the standard formula intensity × (duration − 1) × frequency. Therefore, Pearson correlations were used to examine the associations among the study variables. Means, standard deviations, and correlations are presented in Table 3. The distribution of PA_total was also examined. In the present sample, PA_total ranged from 0 to 100, with a mean of 34.762 and a standard deviation of 30.669. Based on the conventional PARS-3 classification criteria, 199 participants (44.2%) were classified as having low physical activity (≤19), 77 participants (17.1%) as having moderate physical activity (20–42), and 174 participants (38.7%) as having high physical activity (≥43). These categories were reported only to describe the sample distribution and were not used as predictors in the correlation or SEM analyses.

**Table 3:** Means and correlations among variables.

Variable	1	2	3	4	5	6	7	8	M	SD
1. PA_total	1								34.762	30.669
2. IR	−0.302^***^	1							2.534	0.817
3. DR	0.225^***^	−0.328^***^	1						2.908	0.819
4. Mindfulness	0.07	0.127^**^	−0.068	1					3.475	1.017
5. IC_cost	0.164^***^	−0.211^***^	0.184^***^	−0.034	1				23.847	9.982
6. WM_RT	0.216^***^	−0.243^***^	0.201^***^	−0.068	0.519^***^	1			930.557	123.364
7. CF_RT	0.303^***^	−0.283^***^	0.247^***^	−0.039	0.520^***^	0.496^***^	1		800.962	88.813
8. EF_RT	0.277^***^	−0.299^***^	0.257^***^	−0.057	0.828^***^	0.818^***^	0.818^***^	1	0	0.821

Note. PA_total = continuous physical activity score calculated using the PARS-3 formula: intensity × (duration − 1) × frequency. The categorical PA level variable was used only to describe the sample distribution and was not used in the correlation, mediation, or moderated mediation analyses. IR = intrusive rumination; DR = deliberate rumination; IC_cost = inhibitory-control interference-cost index derived from the Flanker task; WM_RT = working-memory reaction-time index derived from the 2-back task; CF_RT = cognitive-flexibility reaction-time index derived from the More–Odd Shifting task; EF_RT = reaction-time-based executive-function composite. IC_cost, WM_RT, and CF_RT were standardized and averaged to form EF_RT. These indicators were not reverse-coded. For IC_cost, WM_RT, CF_RT, and EF_RT, lower values indicate shorter reaction times or smaller RT costs and therefore better RT-based task performance. Pearson correlations are reported. *p < .05, **p < .01, ***p < .001.

As shown in Table 3, PA_total was negatively correlated with intrusive rumination (IR; r = −.302, p < .001) and positively correlated with deliberate rumination (DR; r = .225, p < .001). The RT-based executive-function indicators were positively interrelated. Specifically, IC_cost was positively associated with WM_RT (r = .519, p < .001) and CF_RT (r = .520, p < .001), and WM_RT was positively associated with CF_RT (r = .496, p < .001). PA_total was also positively correlated with the RT-based EF indicators and the EF_RT composite. Because lower values on these RT-based indicators indicate shorter reaction times or smaller RT costs, these correlations should be interpreted as associations with RT-based task performance rather than as direct evidence of better EF ability. Mindfulness showed a small positive correlation with IR (r = .127, p < .01), a nonsignificant correlation with DR (r = −.068), and weak nonsignificant correlations with the RT-based EF indicators. These descriptive findings were used to inform the subsequent structural equation modeling analyses.

### 3.4. Gender differences in key variables

Gender differences were examined using independent-samples t tests, with Welch’s correction applied when the assumption of equal variances was violated, for the key variables (Table 4; N = 450; males = 266, females = 184). Males reported higher levels of physical activity than females, as indicated by the continuous PARS-3 total score, t(413.452) = 2.991, p = .003, 95% CI = [2.944, 14.236], d = 0.282. Females scored higher on intrusive rumination (IR) and deliberate rumination (DR), IR: t(368.204) = −2.540, p = .011, 95% CI = [−0.353, −0.047], d = −0.244; DR: t(448) = −2.187, p = .029, 95% CI = [−0.320, −0.017], d = −0.210. Mindfulness did not differ significantly by gender, t(448) = 1.645, p = .100, 95% CI = [−0.030, 0.354], d = 0.158. Gender differences in the RT-based executive-function composite were not statistically significant, EF_RT: t(448) = 1.631, p = .104, 95% CI = [−0.026, 0.283], d = 0.156. Overall, the observed gender differences were small in magnitude.

**Table 4:** Gender differences in key variables (N = 450; males = 266, females = 184).

Variable	Male M (SD)	Female M (SD)	t (df)	p	95% CI	Cohen’s d
PA_total	38.274(31.405)	29.685(28.909)	2.991(413.452)	0.003	[2.944, 14.236]	0.282
IR	2.452 (0.778)	2.652 (0.840)	−2.540 (368.204)	0.011	[−0.353,−0.047]	−0.244
DR	2.845 (0.834)	3.013 (0.784)	−2.187 (448)	0.029	[−0.320, −0.017]	−0.210
Mindfulness	3.541 (0.999)	3.408 (1.039)	1.645 (448)	0.100	[−0.030, 0.354]	0.158
EF_RT	0.052 (0.809)	−0.076 (0.835)	1.631 (448)	0.104	[−0.026, 0.283]	0.156

Note. PA_total = continuous physical activity score calculated using the PARS-3 formula: intensity × (duration − 1) × frequency; IR = intrusive rumination; DR = deliberate rumination; EF_RT = reaction-time-based executive-function composite. Lower EF_RT values indicate better RT-based task performance. Welch’s correction was applied when the assumption of equal variances was violated.

Because significant gender differences were observed for PA_total, IR, and DR, gender was included as a covariate in the subsequent structural models. A one-way ANOVA was conducted to compare 17-, 18-, and 19-year-olds on the key study variables (Table 5). No significant age-group effects emerged for PA_total, intrusive rumination (IR), deliberate rumination (DR), mindfulness, or the RT-based executive-function composite (EF_RT), Fs = 0.206–0.614, ps = .541–.814. All eta squared values were trivially small (η² ≤ .003), indicating negligible practical differences across the three age cohorts.

**Table 5:** One-way ANOVA results across age groups.

Variable	F	p	η²
PA_total	0.614	0.541	0.0027
IR	0.206	0.814	0.0009
DR	0.321	0.725	0.0014
Mindfulness	0.418	0.659	0.0019
EF_RT	0.373	0.689	0.0017

Note. PA_total = continuous physical activity score calculated using the PARS-3 formula: intensity × (duration − 1) × frequency; IR = intrusive rumination; DR = deliberate rumination; EF_RT = reaction-time-based executive-function composite. Lower EF_RT values indicate better RT-based task performance.

### 3.5. *Analysis of indirect associations*

A gender-adjusted parallel mediation model was tested using structural equation modeling with bias-corrected bootstrapping (5,000 resamples) to examine whether intrusive rumination (IR) and deliberate rumination (DR) were involved in indirect associations between physical activity (PA_total) and the RT-based executive-function composite (EF_RT; Table 6). The estimates reported in this section were obtained from the gender-adjusted parallel mediation model without interaction terms, and all coefficients are standardized estimates.

**Table 6:** Parallel mediation effects through rumination.

Path	Effect (β)	Bootstrap 95%CI		Proportion mediated(%)
		Lower	Upper	
PA_total → IR → EF_RT	0.055	0.025	0.096	20.1
PA_total → DR → EF_RT	0.039	0.015	0.073	14.2
Total indirect effect	0.093	0.056	0.143	34.3
Direct effect: PA_total → EF_RT	0.179	0.080	0.278	—
Total effect	0.272	0.180	0.362	100

Note. PA_total = continuous physical activity score calculated using the PARS-3 formula; IR = intrusive rumination; DR = deliberate rumination; EF_RT = reaction-time-based executive-function composite. Lower EF_RT values indicate better RT-based executive-function task performance. Estimates were obtained from the gender-adjusted parallel mediation model without interaction terms. All reported coefficients are standardized estimates. Bias-corrected bootstrap confidence intervals were based on 5,000 resamples. Proportion mediated = indirect effect/ total effect. The total effect reported in this table refers to the unconditional total association between PA_total and EF_RT in the nonmoderated parallel mediation model.

For the IR pathway (PA_total → IR → EF_RT), the indirect association was significant, β = .055, 95% CI [.025,.096], accounting for 20.1% of the total association. Although the PA_total → IR path and the IR → EF_RT path were both negative, their product was positive; therefore, the indirect association through IR is reported as a positive coefficient in relation to EF_RT. For the DR pathway (PA_total → DR → EF_RT), the indirect association was also significant, β = .039, 95% CI [.015,.073], accounting for 14.2% of the total association. The total indirect association through rumination was significant, β = .093, 95% CI [.056,.143], accounting for 34.3% of the total association between PA_total and EF_RT. The direct association between PA_total and EF_RT remained significant, β = .179, 95% CI [.080,.278], and the total association was β = .272, 95% CI [.180,.362].

Because lower EF_RT values indicate better RT-based task performance, the positive direct and total associations between PA_total and EF_RT indicate that higher PA_total was associated with higher RT-based scores, reflecting slower or less efficient RT-based task performance rather than better executive-function ability. The estimates in Table 6 were obtained from the gender-adjusted parallel mediation model without interaction terms; therefore, the total association represents the unconditional total association in the nonmoderated model.

### 3.6. Analysis of moderated mediation

After estimating the gender-adjusted parallel mediation model, we further tested a gender-adjusted observed-variable moderated mediation model. This model differed from the parallel mediation model reported in Table 6 because it included mindfulness and two mean-centered interaction terms: PA_total × mindfulness and IR × mindfulness. Therefore, the coefficients and indirect associations reported in Table 8 should not be expected to be identical to those in Table 6.

Before interpreting the interaction terms, multicollinearity diagnostics were examined. The VIF and tolerance values indicated no serious multicollinearity among the predictors and interaction terms. The moderated mediation model tested whether mindfulness moderated the PA_total → IR path and the IR → EF_RT path. Gender was included as a covariate. Because mindfulness moderated both the PA_total → IR path and the IR → EF_RT path, the moderated mediation pattern was summarized using two path-specific component indices rather than a single overall linear index. As shown in Table 7, the a-path component index was 0.032, with a 95% bias-corrected bootstrap CI of [0.011, 0.059], indicating that mindfulness moderated the PA_total → IR component of the indirect association. The b-path component index was −0.076, with a 95% bias-corrected bootstrap CI of [−0.114, −0.043], indicating that mindfulness moderated the IR → EF_RT component of the indirect association. The complete conditional indirect associations, calculated as the product of the two moderator-dependent slopes, are reported in Table 8.

**Table 7:** Path-specific component indices for the moderated mediation model.

Path	Path-specific component index	95% CI (LL, UL)	Decision
PA_total → IR → EF_RT (a-path moderation)	0.032	[0.011, 0.059]	Significant
PA_total → IR → EF_RT (b-path moderation)	−0.076	[-0.114, -0.043]	Significant

Note. PA_total = continuous physical activity score calculated using the PARS-3 formula; IR = intrusive rumination; EF_RT = reaction-time-based executive-function composite. Lower EF_RT values indicate better RT-based task performance. Gender was included as a covariate. Bias-corrected bootstrap confidence intervals were based on 5,000 resamples. The values in this table are path-specific component indices rather than full conditional indirect effects; the full conditional indirect effects are presented in Table 8.

**Table 8:** Conditional indirect effects of PA_total on EF_RT through intrusive rumination at different levels of mindfulness.

Mindfulness level	PA_total → IR slope	IR → EF_RT slope	Conditional indirect effect (β)	Bootstrap 95% CI Lower	Bootstrap 95% CI Upper	Decision
−1 SD (low)	−0.113	−0.420	0.047	−0.009	0.112	Not significant
Mean	−0.302	−0.169	0.051	0.022	0.091	Significant
+1 SD (high)	−0.491	0.081	−0.040	−0.121	0.033	Not significant

Note. PA_total = continuous physical activity score calculated using the PARS-3 formula; IR = intrusive rumination; EF_RT = reaction-time-based executive-function composite. Lower EF_RT values indicate better RT-based executive-function task performance. Conditional indirect effects were estimated from the gender-adjusted observed-variable moderated mediation model, in which PA_total × mindfulness and IR × mindfulness were entered as mean-centered product terms. All conditional slopes and conditional indirect effects are standardized estimates. Bootstrap confidence intervals were based on 5,000 resamples. For each bootstrap sample, a(W) and b(W) were recomputed and multiplied to obtain the bootstrap distribution of the conditional indirect effect. Because the indirect association through IR was conditional on mindfulness, no single unconditional total indirect effect or total effect is reported for this moderated mediation model.

As summarized in Fig 1, PA_total was negatively associated with IR (β = −.314, p < .001) and positively associated with DR (β = .251, p < .001). IR was negatively associated with EF_RT (β = −.207, p < .001), whereas DR was positively associated with EF_RT (β = .156, p < .01). The direct association between PA_total and EF_RT remained significant (β = .275, p < .001). Mindfulness significantly moderated both the PA_total → IR path (β_int = −.193, p < .001) and the IR → EF_RT path (β_int = .301, p < .001). Because lower EF_RT values indicate better RT-based task performance, EF_RT-related paths should not be interpreted as positively scored EF ability effects.

The conditional indirect effects reported in Table 8 were obtained from the gender-adjusted observed-variable moderated mediation model, in which PA_total × mindfulness and IR × mindfulness were entered as mean-centered product terms. All conditional slopes and conditional indirect effects are reported as standardized estimates. The conditional indirect association between PA_total and EF_RT through IR varied across levels of mindfulness (Table 8). At the mean level of mindfulness, the conditional indirect effect was significant, β = .051, 95% CI [.022,.091]. At low mindfulness (−1 SD), the conditional indirect effect was positive but not statistically significant, β = .047, 95% CI [−.009,.112]. At high mindfulness (+1 SD), the conditional indirect effect was negative but not statistically significant, β = −.040, 95% CI [−.121,.033]. Importantly, the positive IR → EF_RT slope at high mindfulness (β = .081) should not be interpreted as evidence that intrusive rumination was associated with better executive function. Because EF_RT is a reaction-time-based composite in which lower values indicate better task performance, a positive IR → EF_RT slope indicates an association with higher EF_RT values, reflecting longer reaction times or larger RT costs. Moreover, the conditional indirect effect at high mindfulness was not statistically significant, so this pattern should be interpreted cautiously.

**Fig 2:**
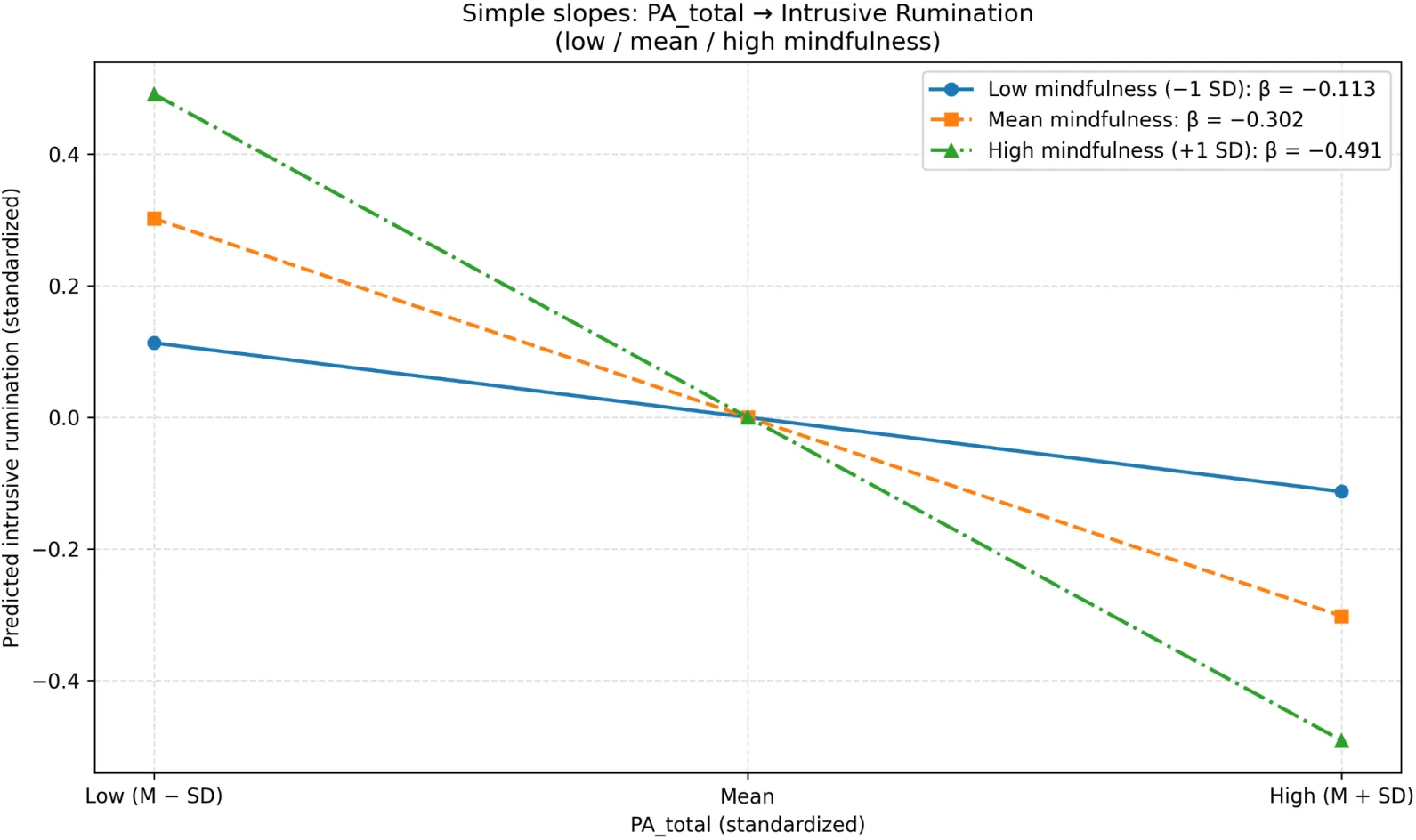
Simple slopes for the association between PA_total and intrusive rumination at low (−1 SD), mean (M), and high (+1 SD) levels of mindfulness.

The simple-slope plots showed a consistent moderation pattern for the PA_total → IR pathway. Specifically, the negative association between PA_total and intrusive rumination (IR) was weakest at low mindfulness (β = −.113), stronger at the mean level of mindfulness (β = −.302), and strongest at high mindfulness (β = −.491). This pattern indicates that the negative association between PA_total and IR became stronger as mindfulness increased (see Fig 2).

**Fig 3:**
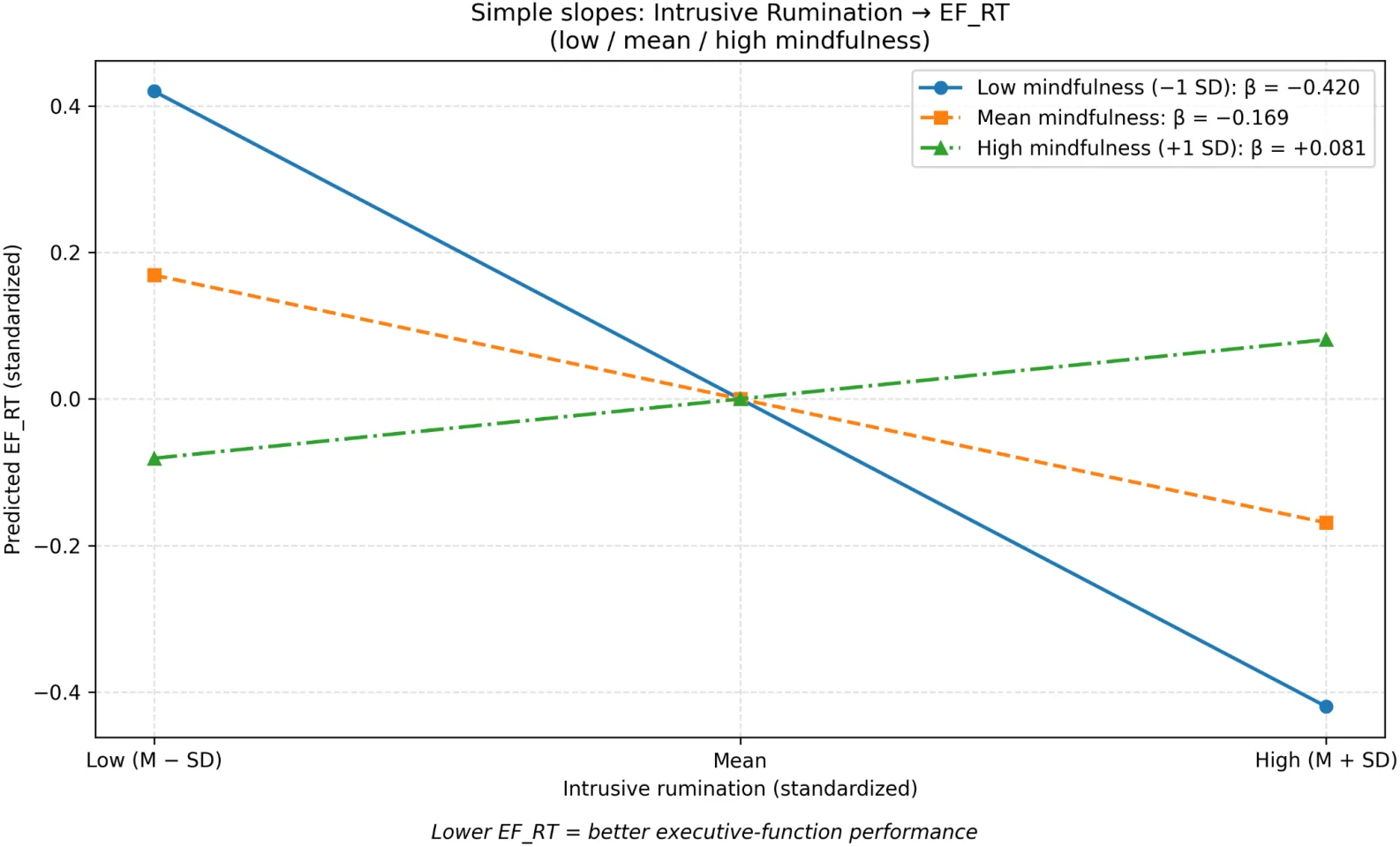
Simple slopes for the association between intrusive rumination and EF_RT at low (−1 SD), mean (M), and high (+1 SD) levels of mindfulness. EF_RT = reaction-time-based executive-function composite; lower EF_RT values indicate better RT-based task performance.

For the IR → EF_RT pathway, the negative association was strongest at low mindfulness (β = −.420), weaker at the mean level of mindfulness (β = −.169), and reversed slightly at high mindfulness (β = .081). This pattern suggests that the association between intrusive rumination and EF_RT varied across levels of mindfulness and became progressively weaker as mindfulness increased (see Fig 3).

## 4. Discussion

Before discussing the findings, it is important to note that the present study used a cross-sectional design. Therefore, the observed mediation and moderation patterns should be interpreted as associations rather than evidence of causal or temporal pathways. Although the tested models were theoretically informed, the data do not establish whether physical activity precedes changes in rumination or EF_RT, or whether rumination temporally explains the association between physical activity and EF_RT. The following discussion therefore focuses on possible interpretations of the observed associations, which should be further examined in longitudinal and experimental studies.

### 4.1. Gender- and age-related differences

The descriptive analyses showed small but significant gender differences in PA_total, IR, and DR. Males reported higher PA_total than females, whereas females reported higher levels of both intrusive and deliberate rumination. These findings are broadly consistent with previous evidence showing that adolescent boys often report higher physical activity levels, while girls tend to show higher levels of repetitive negative thinking and rumination [50–52]. In contrast, gender differences in mindfulness and EF_RT were not statistically significant, suggesting that these variables were relatively comparable across gender groups in the present sample.

Age-group comparisons among 17-, 18-, and 19-year-olds showed no significant differences in PA_total, rumination, mindfulness, or EF_RT. This may partly reflect the restricted age range of the sample and the relative developmental proximity of late adolescents in a university context. Previous developmental work suggests that core executive functions show greater consolidation by late adolescence, although individual differences and task-specific refinements may remain [5,53]. Because gender differences were observed for several key variables, gender was included as a covariate in the subsequent structural models.

### 4.2. Direct association between physical activity and EF_RT

The present study found that PA_total was significantly and positively associated with EF_RT after accounting for rumination pathways and gender. This direction was unexpected because much of the PA–EF literature has reported beneficial associations between physical activity and executive-function outcomes in children and adolescents [5,8,9]. In the present study, however, EF_RT is a reaction-time-based composite in which lower values indicate better RT-based task performance. Therefore, the positive association between PA_total and EF_RT indicates that higher PA_total was associated with higher RT-based scores, reflecting slower or less efficient RT-based task performance rather than better executive-function ability.

This unexpected direction should be considered in light of how both PA and EF were operationalized in the present study. PA_total was a global self-reported score that reflected overall physical activity amount but did not distinguish activity type, cognitive engagement, timing, fatigue, recovery state, or contextual factors. These factors may influence RT-based EF performance in different directions. For example, cognitively engaging activity may relate differently to EF_RT than repetitive or fatigue-inducing activity, and acute exercise timing may also influence reaction-time performance [54]. In addition, EF_RT was a composite of RT-based indicators rather than an accuracy-based or positively scored EF ability measure. Therefore, this finding should be interpreted as an unexpected cross-sectional association specific to the present measurement approach, rather than as evidence that physical activity impairs executive function.

### 4.3. Indirect associations via intrusive and deliberate rumination

The parallel mediation model showed that both IR and DR were involved in significant indirect associations between PA_total and EF_RT. These findings suggest that rumination may be a relevant psychological process in the relationship between physical activity and RT-based executive-function performance. However, because the data were cross-sectional, these results should be interpreted as indirect associations rather than causal mediation pathways.

For the IR pathway, PA_total was negatively associated with IR, and IR was negatively associated with EF_RT. This pattern is not straightforward because intrusive rumination is usually conceptualized as involuntary and repetitive negative thinking that may interfere with attentional control and working-memory processes [17, 22]. Given that lower EF_RT values indicate better RT-based task performance, the negative IR → EF_RT association should not be interpreted as evidence that intrusive rumination improves executive function. Instead, it may reflect the complexity of RT-based performance, including response strategy, arousal, task engagement, or other unmeasured individual differences. Thus, the IR pathway should be interpreted as a statistical association pattern that requires further testing with longitudinal designs and more fine-grained EF indicators.

The DR pathway also showed a significant indirect association. DR is often conceptualized as a more reflective and goal-oriented form of cognitive processing, which may be related to meaning-making, problem analysis, and self-regulation [21, 55]. Nevertheless, the present finding should not be interpreted as showing a clearly beneficial pathway. In this study, higher PA_total was associated with higher DR, and higher DR was associated with higher EF_RT values. Because higher EF_RT reflects longer reaction times or larger RT costs, this association may indicate that deliberate rumination is linked to more effortful, reflective, or slower responding under some task conditions. Future studies should examine whether DR is associated with faster, slower, or more accurate EF performance under different task demands, cognitive-load conditions, and levels of constructive versus over-elaborative reflection.

### 4.4. Conditional associations involving mindfulness

The moderated mediation results suggest that mindfulness conditioned the associations among PA_total, IR, and EF_RT. Specifically, the negative association between PA_total and IR was stronger at higher levels of mindfulness. Mindfulness is commonly understood as present-moment awareness with an accepting and nonjudgmental orientation toward internal and external experiences [33, 34]. From this perspective, adolescents with higher mindfulness may process physical-activity-related bodily or affective experiences with less elaborative negative thinking. This may help explain why PA_total was more strongly associated with lower IR among adolescents with higher mindfulness. However, because post-exercise affective processing, interoceptive awareness, and cognitive reactivity were not directly assessed, this explanation should be viewed as theoretical rather than directly demonstrated by the present data.

Mindfulness also moderated the association between IR and EF_RT. The simple-slope results indicated that the negative association between IR and EF_RT was strongest at low mindfulness, weaker at the mean level of mindfulness, and slightly reversed at high mindfulness. This pattern suggests that the association between intrusive rumination and RT-based executive-function performance differed across levels of mindfulness. Theoretically, mindfulness may reduce the extent to which intrusive thoughts develop into persistent negative cognitive cycles by promoting decentering, reperceiving, and reduced cognitive reactivity [35,39,40]. Nevertheless, the observed interaction should be interpreted as a conditional association rather than evidence that mindfulness causally buffers the effect of intrusive rumination on executive function.

The small positive IR → EF_RT slope observed at high mindfulness should be interpreted with particular caution. Because EF_RT is scored such that lower values reflect better RT-based performance, the positive slope does not indicate that intrusive rumination was associated with better executive function. Instead, it suggests a weak association with higher RT-based scores. One possible theoretical explanation is that adolescents with higher mindfulness may process intrusive thoughts differently from those with lower mindfulness. Rather than becoming automatically elaborated into persistent negative cognitive cycles, intrusive thoughts may be noticed with greater decentering and nonjudgmental awareness. This altered mode of processing may weaken or change the association between IR and RT-based task responding. However, the conditional indirect effect at high mindfulness was not statistically significant; therefore, this explanation remains speculative and should not be treated as evidence that intrusive rumination is beneficial among highly mindful adolescents. Future studies should test whether this pattern replicates and whether highly mindful adolescents process intrusive thoughts differently in ways that can be observed directly.

### 4.5. Implications and future directions

The present findings suggest that psychological factors should be considered when examining the association between physical activity and RT-based EF outcomes in late adolescents. In particular, distinguishing between intrusive and deliberate rumination may help characterize why the PA_total–EF_RT association did not conform to a straightforward beneficial PA–EF pattern.

These findings may also inform future intervention research, but they should not be interpreted as direct evidence for intervention effectiveness. Because no intervention was conducted and the data were cross-sectional, practical applications should remain tentative. Future studies could examine whether physical activity programs, especially those involving cognitively engaging activities, are associated with changes in rumination and RT-based executive-function performance. They could also test whether mindfulness-based components are associated with changes in the link between physical activity and intrusive rumination. These questions should be examined using longitudinal, randomized, or experimental designs that combine objective physical-activity measures with EF indicators incorporating both reaction time and accuracy.

## 5. Conclusions

This study examined the associations among PA_total, rumination, mindfulness, and RT-based executive-function performance in late adolescents. PA_total showed a positive direct association with EF_RT and indirect associations involving intrusive and deliberate rumination. Because higher EF_RT values indicate longer reaction times or larger RT costs, the positive PA_total–EF_RT association suggests that higher PA_total was related to slower or less efficient RT-based task performance in the present sample. This unexpected direction should be interpreted cautiously and does not indicate that physical activity impairs executive function. Mindfulness conditioned the PA_total → IR and IR → EF_RT associations, suggesting that the IR-related indirect association differed across levels of mindfulness. Because EF_RT is a reaction-time-based composite, coefficients involving EF_RT should be interpreted in terms of RT-based task performance rather than as positively scored EF ability effects. Overall, the findings highlight rumination and mindfulness as psychological factors that may help characterize the association between physical activity and RT-based executive-function performance. Given the cross-sectional design, these findings should be interpreted as associations rather than evidence of causal mechanisms or intervention effects.

## 6. Limitations and Future Directions

Several limitations should be considered when interpreting the findings. First, the study used a cross-sectional design and convenience sampling from a limited regional context. Therefore, the observed mediation and moderated mediation patterns should be interpreted as associations rather than evidence of causal or temporal pathways. Future longitudinal and experimental studies are needed to clarify the temporal ordering and potential bidirectionality among physical activity, rumination, mindfulness, and RT-based executive-function performance.

Second, physical activity was assessed using the self-reported PARS-3 rather than objective measures such as accelerometry. Although the continuous PARS-3 total score was used in the main analyses and the categorical PA classification was retained only for descriptive purposes, self-report measures may still be affected by recall bias and reporting error. Future studies should include objective physical-activity indicators and examine whether the present associations remain stable across different measurement methods.

Third, executive function was indexed using RT-based indicators from three computerized tasks and summarized as the EF_RT composite. Although task accuracy was recorded and reported descriptively, accuracy-based indicators were not incorporated into the main SEM models. Therefore, potential speed–accuracy trade-offs cannot be fully ruled out. In addition, the EF_RT composite provides a broad summary of RT-based task performance but may obscure domain-specific patterns in inhibitory control, working memory, and cognitive flexibility. Future studies should include multiple indicators for each EF domain and integrate both RT and accuracy-based measures when constructing EF outcomes.

Fourth, rumination was assessed using the C-ERRI without asking participants to anchor their responses to a specific stressful or traumatic event. Therefore, the IR and DR scores should be interpreted as ERRI-based indicators of recent ruminative thinking rather than as event-specific post-traumatic rumination. This may raise a construct-validity concern and limit comparability with studies using the ERRI in its original event-specific context. Future studies in non-clinical adolescent samples should consider using rumination measures designed for general, non-event-specific rumination or should provide an explicit event anchor when using the ERRI.

Finally, although gender was included as a covariate and age-group differences were examined, other potentially relevant factors were not fully assessed or controlled. These may include sleep quality, academic stress, socioeconomic background, physical fitness, psychological symptoms, and the type, intensity, timing, or context of physical activity. Future research should incorporate a broader range of covariates, objective behavioral measures, and more diverse samples to clarify for whom and under what conditions physical activity, rumination, mindfulness, and RT-based executive-function performance are most closely related.

## Supporting information

S1 Table Accuracy of Executive Function Tasks. (DOCX)

S2 Table Common latent factor analysis for common method variance. (DOCX)
