# Supplementary material for: Physical activity and executive function in adolescents: Dual rumination pathways and mindfulness moderation
Source: PLoS One. 2026 Sep 1;21(9):e0357384. doi: 10.1371/journal.pone.0357384 (PMC13533373; doi:10.1371/journal.pone.0357384)
Supplement: Supplementary file 1 [file pone.0357384.s001.docx]

Supplementary Material (Table S1)

| Table S1. Accuracy of Executive Function Tasks | |
| --- | --- |
| Task (Domain) | Accuracy (%) (Mean ± SD) |
| Flanker task | **96.84 ± 3.72** |
| 2-back task | **81.93 ± 11.88** |
| More–Odd Shifting task | **93.76 ± 6.48** |

Note. Accuracy represents the percentage of correct responses for each executive-function task. For the Flanker and More–Odd Shifting tasks, accuracy values were averaged across task conditions. Accuracy rates are reported as descriptive indicators of task performance and were used to evaluate whether the RT-based EF indicators were interpreted under acceptable accuracy conditions. RT-based indicators were retained as the primary EF measures in the main analyses.
