# Supplementary material for: Physical activity and executive function in adolescents: Dual rumination pathways and mindfulness moderation
Source: PLoS One. 2026 Sep 1;21(9):e0357384. doi: 10.1371/journal.pone.0357384 (PMC13533373; doi:10.1371/journal.pone.0357384)
Supplement: Supplementary file 2 [file pone.0357384.s002.docx]

Supplementary Table S2. Common latent factor analysis for common method variance

| **Model** | **χ²/df** | **RMSEA** | **SRMR** | **CFI** | **TLI** |
| --- | --- | --- | --- | --- | --- |
| Initial measurement model | 1.065 | 0.012 | 0.036 | 0.994 | 0.994 |
| Common latent factor model | 1.042 | 0.010 | 0.034 | 0.997 | 0.996 |
| Absolute change | 0.023 | 0.002 | 0.002 | 0.003 | 0.002 |

Note. CLF = common latent factor; RMSEA = root mean square error of approximation; SRMR = standardized root mean square residual; CFI = comparative fit index; TLI = Tucker–Lewis index. Small changes in model fit after adding the CLF suggested that common method variance was unlikely to substantially distort the measurement model.
